# Medial sleeve fractures in elite‐athletes: A heterogeneous group, anatomical and case‐based considerations

**DOI:** 10.1002/ksa.12489

**Published:** 2024-10-03

**Authors:** Kishan R. Ramsodit, Ruben Zwiers, Miki Dalmau‐Pastor, Vincent Gouttebarge, Mario Maas, Gino M. M. J. Kerkhoffs

**Affiliations:** ^1^ Amsterdam UMC Location University of Amsterdam, Department of Orthopedic Surgery and Sports Medicine Amsterdam The Netherlands; ^2^ Academic Center for Evidence‐based Sports Medicine (ACES) Amsterdam The Netherlands; ^3^ Amsterdam Collaboration for Health and Safety in Sports (ACHSS), IOC Research Center of Excellence Amsterdam The Netherlands; ^4^ Amsterdam Movement Sciences (AMS), Aging & Vitality, Musculoskeletal Health, Sports Amsterdam The Netherlands; ^5^ Department of Orthopedic Surgery, Flevoziekenhuis Almere The Netherlands; ^6^ Human Anatomy and Embryology Unit, Department of Pathology and Experimental Therapeutics, School of Medicine and Health Sciences The University of Barcelona Barcelona Spain; ^7^ MIFAS by GRECMIP (Minimally Invasive Foot and Ankle Society) Merignac France; ^8^ Section Sports Medicine, Faculty of Health Sciences University of Pretoria Pretoria South Africa; ^9^ Amsterdam UMC Location University of Amsterdam Department of Radiology and Nuclear Medicine Amsterdam The Netherlands

**Keywords:** ankle sprain, deltoid ligament, elite athlete, medial ankle instability, medial sleeve, return to performance

## Abstract

**Purpose:**

The purpose of this study is to provide a detailed description of the anatomy and radiology of the medial sleeve and present an approach in its management among elite athletes.

**Methods:**

Five cases of elite athletes who underwent treatment for a medial sleeve injury of which the diagnosis was confirmed through physical examination and additional magnetic resonance imaging scan are described in this study.

**Results:**

Two patients presented with isolated medial sleeve injuries, while the other three patients suffered from concomitant ankle injuries. Non‐operative treatment consisted of relative rest, soft cast immobilization and mobilization in a walking boot or kinesiotape which was successful in four of the cases with regard to the medial sleeve. One patient underwent surgery due to syndesmotic instability. Another patient presented with combined medial and lateral ankle instability which was treated surgically with an open medial and lateral ligament repair. All patients were able to return to their pre‐injury sports and at the time of the last follow‐up were still playing in their pre‐injury level of competition.

**Conclusion:**

Medial sleeve injuries of the ankle in elite athletes should be considered in the differential diagnosis for athletes presenting with medial ankle pain. Inherent knowledge regarding anatomy is essential when guiding the management of these injuries which can be treated successfully with a non‐operative approach consisting of relative rest, immobilization, kinesiotape and physical therapy. In case of persistent medial instability or rotational instability, surgical repair is a viable treatment option. Both modalities allow athletes to return to the pre‐injury level of competition. However, early diagnosis is crucial to minimize the delay of appropriate treatment and avoid potential residual symptoms.

**Level of Evidence:**

Level IV.

**Abbreviations**
AiTFL: anterior inferior tibiofibular ligament
ATFL: anterior talofibular ligament
COVID‐19: coronavirus disease 2019
dDL: deep deltoid ligament
MRI: magnetic resonance imaging
NRS: numerical rating scale
PE: physical examination
sDL: superficial deltoid ligament
WB: weight‐bearing

## INTRODUCTION

Ankle sprains are one of the most common sports injuries, with the deltoid ligament being involved in 5%–15% of all ankle sprains [15, 16, 21]. Despite increasing awareness regarding ankle injuries, the medial collateral ankle ligament (deltoid ligament) continues to receive less attention even though it is the primary ligamentous medial stabilizer of the ankle joint [2, 3]. This might be due to the greater strength of the deltoid ligament and the stability this provides compared to the lateral ankle ligaments. Arthroscopic findings in patients of the general population being treated for chronic lateral ankle instability showed injury to the deltoid ligament in 40%–72% of the cases, demonstrating that the true incidence of deltoid injuries is perhaps underestimated [8, 12]. A previous study evaluating magnetic resonance imaging (MRI) scans after acute ankle sprains in professional athletes observed deltoid injuries in 49% of the cases [19].

**Figure 1:**
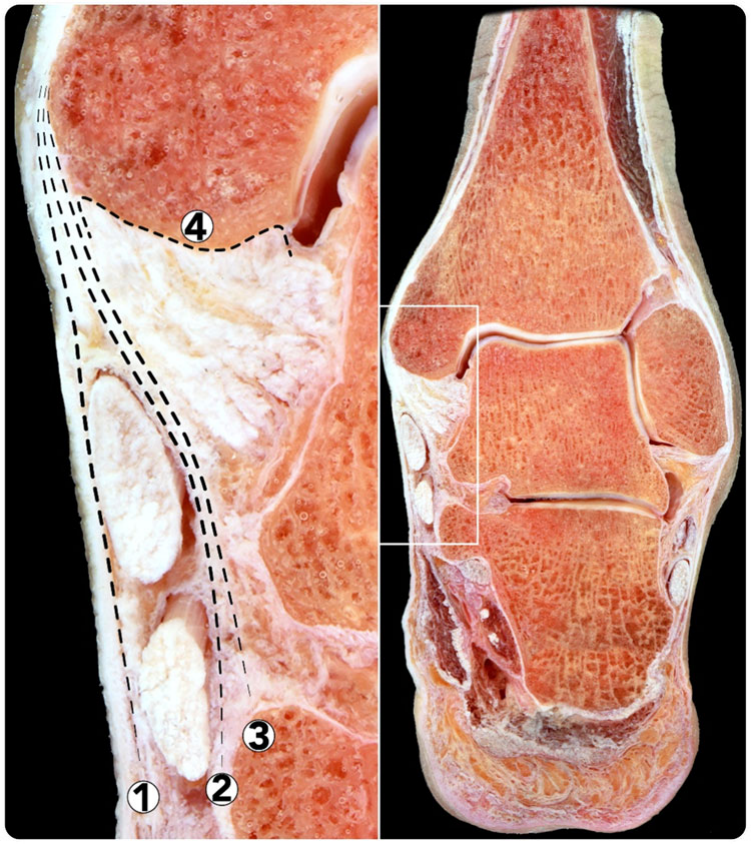
Frontal cross‐section of a specimen, demonstrating the detailed anatomy of the structures inserted on the medial malleolus. 1. Flexor retinaculum. 2 Flexor tendons sheath (flexor digitorum longus and tibialis posterior). 3. Superficial deltoid ligament. 4. Deep deltoid ligament.

Anatomically, a large variance exists regarding the number of components of the deltoid ligament [3]. However, the overall agreement is that it consists of a superficial and a deep layer separated by adipose tissue [4]. Various types of injuries at the origin of the superficial deltoid ligament (sDL) have been described in the current literature, with injury to the medial sleeve of the ankle being relatively unknown [7, 14, 18, 20]. This medial sleeve is a fibrous tissue sheath consisting of, from the anterior to the posterior part of the medial malleolus, the sDL origin extending posteriorly with fibres blending with the periosteum of the medial malleolus and the flexor retinaculum insertion [1, 6, 7]. In fact, the periosteum of the medial malleolus receives insertion of a number of structures, from superficial to deep: the flexor retinaculum, the flexor tendon sheaths (flexor digitorum longus and tibialis posterior), sitting on top and often difficult to differentiate from the sDL, and the deep deltoid ligament (dDL) (Figure 1). While outcomes after injuries to the sDL have been reported, little is known specifically regarding medial sleeve lesions [13].

Therefore, the aim of this study was to (i) provide a detailed description of the anatomy and radiology of the medial sleeve and (ii) present an approach to its management among elite athletes.

### Anatomy of the deltoid ligament

**Figure 2:**
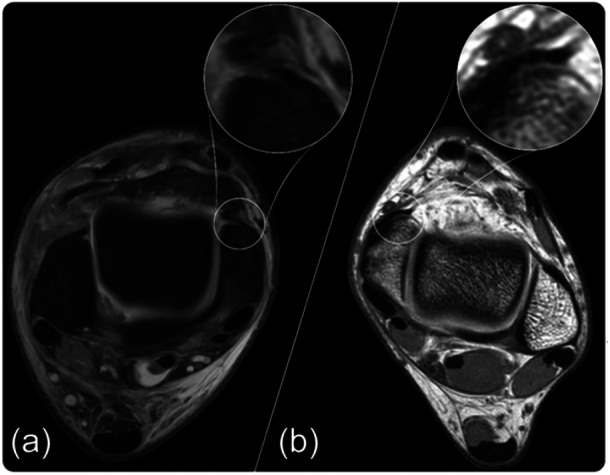
T1 (a) and T2 (b) axial MRI sequence of the ankle joint, showcasing a partial medial sleeve rupture with visible fluid between the anterior portion of the sleeve and medial malleolus.

**Figure 3:**
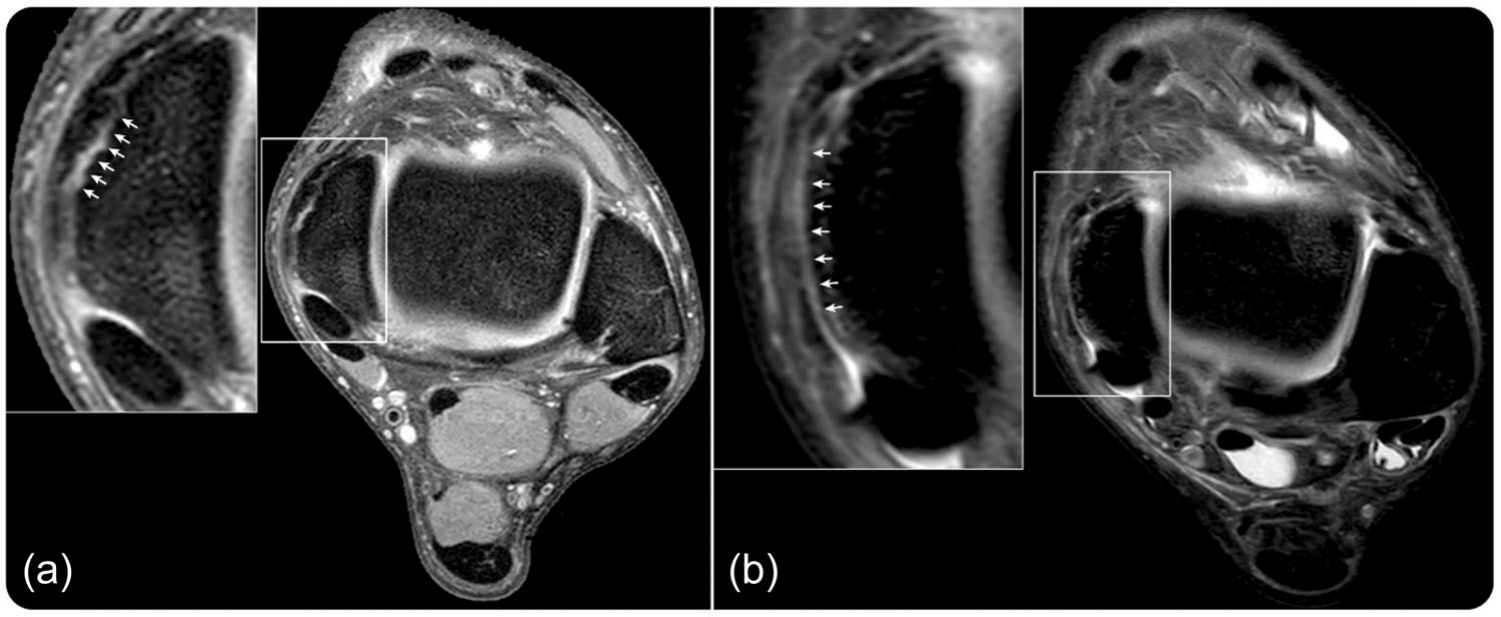
T2 Axial MRI sequence of the ankle joint, showcasing a complete medial sleeve rupture with visible fluid between the sleeve and medial malleolus.

The deltoid ligament is the primary medial stabilizer of the ankle joint with the flexor retinaculum and posterior tibial tendon providing additional stability. The sDL crosses both the tibiotalar and subtalar joint, while the dDL crosses only the tibiotalar joint, each exerting different functions in the stabilization of the ankle joint. The dDL prevents ankle eversion and lateral translation of the talus. It is comprised of the intra‐articular tibiotalar fascicle, which is often divided into an anterior and a posterior part. It originates on the intra‐articular part of the medial malleolus (anterior and posterior colliculus) and inserts on the talus, just below the comma‐shaped medial talar articular surface, also having a comma‐shaped morphology, thus resembling a yin‐and‐yang shape [9]. The sDL resists valgus and external rotation of the talus. It is comprised of, from anterior to posterior, the tibionavicular fascicle, the tibiospring fascicle and the tibiocalcaneal fascicle. The sDL originates at the extra‐articular part of the medial malleolus; as from previous descriptions, the ligament fibres extend posteriorly and blend with the periosteum of the medial malleolus, which in turn blends with the insertion of the flexor retinaculum. This structure, where the origin of the sDL, the periosteum and the origin of the flexor retinaculum merge, is named the ‘fascial sleeve of the medial malleolus’ [7]. A medial sleeve injury entails detachment of the sDL from its origin along with the periosteum and potential involvement of the flexor retinaculum. The extent of the injury is determined by the progression of the stripping lesion from anterior to posterior. Anatomically, three cut‐off patterns can be differentiated: (1) superficial medial sleeve; injury including only the origin of the common sDL origin (Figure 2). (2) Subtotal medial sleeve; injury from the common sDL origin extending to but not involving the flexor retinaculum. (3) Total medial sleeve; injury starting at the common sDL origin extending to and involving the flexor retinaculum entailing a complete sleeve detachment from the medial malleolus (Figure 3).

### Diagnostic evaluation

#### Clinical presentation

At first presentation, it is important to note if there was a distinct trauma mechanism or if the symptoms have emerged gradually. A high index suspicion of medial sleeve injury is warranted in patients presenting with medial ankle pain or an ankle trauma which included an eversion or external rotation element. In the case of gradual‐onset medial ankle pain, damage to the medial sleeve should be considered when there is a feeling of instability on the medial side.

During the physical examination (PE), it is essential to assess swelling and haematoma of the ankle medially, if present. Upon palpation, a distinction can be made between the superficial and deep deltoid by palpating anterior to the medial malleolus and over the medial surface of the medial malleolus approximately 0.5 cm proximal to the inferior border of the medial malleolus.

#### Radiological imaging

MRI scan provided insights into the anatomy of the deltoid ligament to identify which ligamentous structures were damaged and the degree of effusion in the joint. The computed tomography (CT) scan was used to identify potential avulsions, fractures or loose ossicles. For evaluation of the medial sleeve, a high‐resolution axial proton density MRI without fat saturation is advised. Additional axial T2 fat‐saturated images are also helpful. The flexor retinaculum should always be firmly attached to the periosteum/cortex of the medial malleolus. When there is a dehiscence between the cortex and retinaculum with or without accompanying water signals, a medial sleeve injury is present.

### Case presentations

The present study was a retrospective descriptive case series of elite athletes treated for a medial sleeve injury. Ethics approval for this study was provided by the Medical Ethical Committee of Amsterdam UMC (W22_397). The outcome measures for this study were return to training, return to competitive activity and return to performance. Performance was defined as self‐reported pre‐injury level of sport.

#### Considerations on best therapy: Case‐based approach

**Figure 4:**
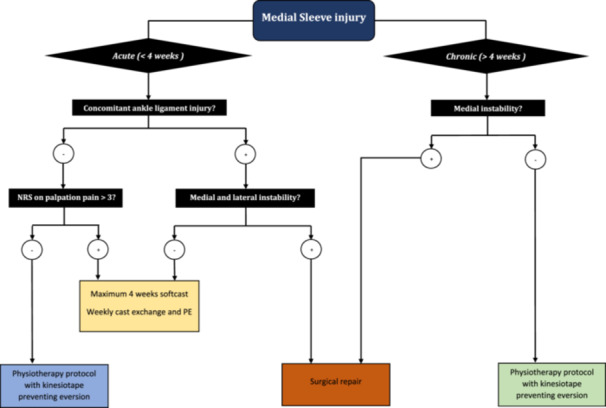
Flowchart of a clinical decision‐making tool when approaching a medial sleeve injury in the elite athlete. NRS, numerical rating scale; PE, physical examination.

The goal of medial sleeve fracture treatment is to ‘close the space’ between the sleeve and medial malleolus to restore a single continuous structure (Figure 4). Non‐operative treatment of medial sleeve injuries can be effective in elite athletes when managed within the acute window (<4 weeks). Options available in the initial non‐operative management are (1) immobilization in a soft cast or (2) kinesiotape preventing eversion movements. The choice of treatment modality is based on joint swelling, level of pain, ability to bear weight and potential concomitant ankle injuries. After an acute medial sleeve injury, there is often visible joint swelling, while the MRI scan shows a visible line of fluid between the medial sleeve and the medial malleolus on the MRI scan. As the body should be able to heal naturally, a 2‐week period of soft cast immobilization or mobilization in a walking boot should be sufficient to allow for adequate rest of the ankle and healing. A period of soft cast immobilization is particularly indicated when there is concomitant ligamentous injury. Weekly cast changes to prevent stiffness as well as PEs are then conducted to evaluate the progress. When the pain level is under control, the athlete is advised to first build up the ankle range of motion, then the strength of the ankle flexors and extensors, and only thereafter focus on balance and proprioception. When running is introduced into the rehabilitation protocol, kinesiotape should be applied preventing eversion movements of the ankle while running is only allowed in straight directions. When comfortable with this, the athlete is allowed to start rotational movements with the ankle without any reaction of the ankle.

If the medial sleeve injury has existed for a prolonged period, the treatment modality is slightly altered. The amount of fluid between medial sleeve and medial malleolus is decreased and there is less swelling of the ankle. Therefore, a prolonged period of soft cast immobilization is not indicated. In these cases, the sleeve injury persists due to inadequate initial treatment. This can be treated with one week of soft cast immobilization or a 2‐ to 3‐week period of relative rest. Thereafter, the athlete is encouraged to start rehabilitation of the affected ankle with kinesiotape preventing eversion introduced during straight runs.

#### Surgical treatment

Surgery is not routinely indicated for medial sleeve injuries. However, in cases of combined medial and lateral ankle instability, as seen in Case 3, surgery is the preferred course of treatment. This provides assured stability with less chance of residual instability. Post‐operatively, the patient receives non‐weight‐bearing (WB) immobilization for two weeks. Subsequently, a 4‐week period of progressive WB and mobilization in a walking boot is introduced.

## RESULTS

Five patients (one female and four males) are presented in this study (Table 1). The median age was 23 years (interquartile range = 20–26). The median clinical follow‐up was 3 months. Three athletes were engaged in professional football, while one athlete was a professional basketball player and the other an international rugby player. Four out of five patients received non‐operative treatment with one of those four (Case 5) needing surgery due to syndesmotic instability. In Case 1, the athlete was treated the day after appearance of symptoms with mobilization in a walking boot for 3 weeks and did not experience any residual symptoms upon his return to performance. He was able to participate in normal training 5 weeks after the injury, played his first match after 6 weeks and returned to performance after 7 weeks. One patient (Case 3) underwent surgery for the medial sleeve due to combined medial and lateral ankle instability. While he returned to playing competitively at his previous level, the timeline in which this occurred was distorted due to the rehabilitation occurring during the coronavirus disease 2019 (COVID‐19) pandemic when group training at football clubs and matches were not possible. All patients returned to their respective pre‐injury sports and at the time of data collection were still competing in their pre‐injury level of competition (Table 1).

**Table 1:** Overview of the demographic, injury, clinical and treatment characteristics with the corresponding return to performance outcomes.

Case	Sex	Age at presentation, years	Pre‐injury sports	Trauma mechanism	Duration of symptoms, *n* days	Diagnosis	Treatment	Return to training, *n* weeks	Return to sport, *n* weeks	Return to performance, *n* weeks
1	M	20	Professional Football Player	Hyper dorsiflexion and anterior translation	1	Total medial sleeve	3 weeks walking boot	5	6	7
2*	M	28	Professional Football Player	Eversion	95	Chronic total medial sleeve	Training build‐up with eversion kinesiotape	N/A	N/A	N/A
3	M	23	Professional Football Player	Combined inversion and eversion	11	Total ATFL injury, Total CFL rupture, total medial sleeve	Medial and lateral ankle ligament repair	25	27	27
4	F	26	International Rugby Player	Fall of multiple opponents on the ankle	5	Subtotal AiTFL rupture, subtotal ATFL rupture, subtotal medial sleeve	4 weeks of soft cast immobilization	11	13	13
5	M	23	National Basketball Player	Dorsiflexion and exo‐rotation	1	Total AiTFL rupture, partial ATFL rupture, partial CFL rupture, subtotal medial sleeve	4 weeks of soft cast immobilization followed by needle arthroscopic inspection of the ankle joint with suture button fixation of the ankle syndesmosis	20	26	N/A

*Note*: *No return to sport data available for the athlete as he was actively participating in training sessions and matches at the time of presentation with discomfort.

Abbreviations: AiTFL, anterior inferior tibiofibular ligament; ATFL, anterior talofibular ligament; CFL, calcaneofibular ligament.

## DISCUSSION

The main finding of this study was that non‐operative treatment of medial sleeve injuries of the ankle in elite athletes is effective with athletes being able to return to pre‐injury level of competitive activity. In case of combined medial and lateral ankle instability, repair of the medial sleeve combined with lateral ligament repair may be warranted to avoid any residual complaints of instability.

### Diagnosis, tips from the radiologist and treatment

Little is known regarding the medial sleeve injuries of the ankle in the current literature. Nonetheless, a distinction between deltoid injuries and medial sleeve injuries is necessary due to the difference in injury pattern based on the anatomy and its possible implications on the optimal treatment strategy and thus the performance of the athlete. Deltoid ligament injuries are commonly graded by the extent of the damage by way of discontinuity, injury location, that is, superficial or deep, anatomic location of the tear relative to osseous structures [5, 16]. While a medial sleeve injury involves the sDL, there is no discontinuity of the ligamentous structure; instead, the sleeve is stripped from the medial malleolus. To the best of the authors' knowledge, the present study was the first of its kind to describe clinical outcomes for this type of injury in elite athletes. A previous radiological study retrospectively analyzed the MRI imaging of patients in the general population who underwent surgery for medial ankle instability and described the medial sleeve injuries [7]. However, diagnosing the medial sleeve injury beforehand in recent years might be aided by the development of higher quality MRI images through which more detailed structures are available for interpretation by the radiologist. Yet, knowledge of this type of lesion and a high index suspicion of the radiologist is needed when judging the radiological imaging. When extensive or multi‐ligamentous injury is present, radiologists need to be wary of satisfaction of search.

In their study, Crim and Longenecker et al. did not state if a non‐operative treatment plan was initially followed [7]. In our study, we have found various approaches to be useful during the non‐operative treatment of this injury. During the acute phase of this injury, we consider rest in the area of the medial ankle structures essential for the healing process. This can be facilitated by a relative rest period, mobilization in a walking boot or an immobilization period with a soft cast. The soft cast immobilization helps to reduce the swelling of the ankle and promote healing. A possible alternative to soft cast immobilization is the walking boot. Although it does not provide significant compression compared to the soft cast, it is adequate in providing rest to an ankle which has little to no swelling. Interestingly, even athletes with multi‐ligamentous ankle injuries (Cases 4 and 5) who were initially treated with a period of soft cast immobilization, reported a vast improvement in palpation pain on the medial sleeve over time. Surgery was therefore not indicated for the deltoid ligament and athletes did not report any residual complaints of the medial ankle during their rehabilitation. Depending on the ankle function, kinesiotape may be provided as a protective aid during the rehabilitation and as secondary prevention. By taping the ankle to prevent eversion movements, the medial ankle structures receive more support and thus protect the athlete during their rehabilitation and initial return to sports activities. While the preferred operative technique in our study was an open approach, previous studies have reported after arthroscopic medial and lateral ligament reconstruction for rotational ankle instability as well [10, 17, 20]. The study by Vega et al. [20] described an ‘open book’ tear of the deltoid ligament, which entailed the separation of the anterior part of the dDL from the medial malleolus during internal rotation of the talus. While the deep fascicle of the deltoid ligament is an intra‐articular structure as seen with an arthroscopic view, the superficial deltoid is not [11]. However, the pathophysiology of the medial sleeve injury shows visible fluid entering the space between the sleeve and medial malleolus which suggests an intra‐articular component through which the fluid originates. This might originate from the anterior part of the tibionavicular fascicle of the superficial deltoid which has been found to lay in an intra‐articular position [9].

### Return to performance: Expectations for the elite athlete

Isolated deltoid ligament injuries are rather uncommon as those more often occur in combination with an ankle fracture or syndesmotic injury. Nonetheless, a distinction between deltoid injuries and medial sleeve injuries is necessary due to the difference in injury pattern based on the anatomy and the possible implications of this on the optimal treatment strategy and thus the performance of the athlete. Due to a lack of literature regarding non‐operative treatment of isolated deltoid injuries in elite athletes, a comparison of clinical course and time lost to injury is not possible. In the presented cases, it is important to note that the athlete with an isolated medial sleeve injury who presented with a chronic injury was still able to be treated non‐operatively. This athlete did not report time away from the sport due to injury and was actively participating in training and playing matches with the kinesiotape. However, adequate treatment in the initial phase can potentially prevent residual complaints hampering athletes' (return to) performance. While three of the remaining four and four out of five athletes returned to performance, all athletes were objectively participating at the pre‐injury level of competition at the time of data collection. This discrepancy between return to competitive activity and return to performance is attributed to the subjective definition of performance in this study. It is hypothesized that in case of an isolated medial sleeve injury treated non‐operatively, an athlete may be able to return to competitive activity between 8 and 10 weeks after injury. However, a larger sample size is needed to adequately evaluate this timeline. When concomitant injury is present, the return to sport timeline is dependent on the recovery of the additional injuries.

## CONCLUSION

Medial sleeve injuries of the ankle in elite athletes should be considered in the differential diagnosis for athletes presenting with medial ankle pain. Inherent knowledge regarding anatomy is essential when guiding the management of these injuries which can be treated successfully with a non‐operative approach consisting of relative rest, immobilization, kinesiotape and physical therapy. In case of persistent medial instability or rotational instability, surgical repair is a viable treatment option. Both modalities allow athletes to return to pre‐injury level of competition. However, early diagnosis is crucial to minimize the delay of appropriate treatment and avoid potential residual symptoms. For future research, evaluation of a larger cohort of patients with a medial sleeve injury is essential to gain a better understanding of the optimal treatment strategy.

## AUTHOR CONTRIBUTIONS

Kishan R. Ramsodit and Ruben Zwiers have made substantial contributions to conception and design, acquisition of data, analysis and interpretation of data and have been involved in drafting the manuscripts. Miki Dalmau‐Pastor, Vincent Gouttebarge, Mario Maas and Gino M. M. J. Kerkhoffs have been involved in revising the manuscript, critically for important intellectual content and have given final approval of the version to be published.

## CONFLICT OF INTEREST STATEMENT

The authors declare no conflict of interest.

## ETHICS STATEMENT

Ethics approval for this study was provided by the Medical Ethical Committee of Amsterdam UMC (W22_397).

## Data Availability

No additional data are available.
